# The millipede genus *Globanus* Attems, 1914, endemic to São Tomé and Príncipe, with the description of a new species (Diplopoda, Spirostreptida, Spirostreptidae)

**DOI:** 10.3897/zookeys.930.49236

**Published:** 2020-04-28

**Authors:** Didier VandenSpiegel, Rowland M. Shelley, Sergei I. Golovatch

**Affiliations:** 1 Royal Museum for Central Africa, Biological Collection and Data Management Unit, B-3080 Tervuren, Belgium; 2 Institute for Problems of Ecology and Evolution, Russian Academy of Sciences, Leninsky pr. 33, Moscow 119071, Russia

**Keywords:** Africa, Diplopoda, Gulf of Guinea, key, *Lobogonus*, taxonomy

## Abstract

During a soil zoological expedition to São Tomé and Príncipe in 2010 by the California Academy of Sciences, millipedes of the genus *Globanus* were collected. Samples of *G.marginescaber* (Karsch, 1884) and *G.integer* (Karsch, 1884) were recovered in addition to those containing a new species. *Globanusdrewesi***sp. nov.** is described and additional records, illustrations, and descriptive notes are given for the other two species. A key to all three species of the genus is provided, and a distribution map is presented. The monotypic genus *Lobogonus* Demange, 1971, which includes *L.trilobatus* Demange, 1971, from Sierra Leone, mainland western Africa, is revalidated and removed from synonymy under *Globanus*. *Lobogonus* is illustrated from a type specimen.

## Introduction

São Tomé and Príncipe are two volcanic islands in the Gulf of Guinea straddling the Equator ca 250 km west off the coast of Gabon. Together with Annobon and Bioko, they belong to the Cameroon volcanic chain. These are classical oceanic islands long known for their peculiar biota. Although the bird fauna is relatively well documented (Christy and Clarke 1998) and a flora exists since the 1920s (Liberato and Espirito Santo 1972–1982) that describes the major elements of the islands’ botany, many other important animal groups remain badly understudied, with Diplopoda, or millipedes, being one of them.

The first diplopods recorded from São Tomé and Príncipe were two species, Spirostreptus (Nodopyge) integer Karsch, 1884, and Spirostreptus (Nodopyge) marginescaber Karsch, 1884, described as new by Karsch (1884) in anecdotal “descriptions” accompanied by no illustrations whatsoever. Later, *S.integer* was relegated to the new genus *Globanus* Attems, 1914, while *S.marginescaber* was considered as a species *incertae sedis* because its holotype was a female (Attems 1914). Spelda (1993) summarized the myriapod fauna of São Tomé Island, reporting six centipede and five millipede species. Amongst the Diplopoda, only two species of Spirostreptidae were considered indigenous: *G.integer* and *G.marginescaber*. All others were likely introductions, either pantropical: *Paraspiroboluslucifugus* (Gervais, 1836) (Spirobolida, Spirobolellidae) and *Orthomorphacoarctata* (de Saussure, 1860) (Polydesmida, Paradoxosomatidae) or widespread and western African: *Teloidenopussulcatus* (Voges, 1878) (Spirostreptida, Spirostreptidae). A number of spirostreptid samples remained unidentified, but Spelda (1993) assigned them all unequivocally to *Globanus*. The only hitherto known diplopod truly endemic to Príncipe seems to be *Monachodesmusfeae* Silvestri, 1927, the type species of *Monachodesmus* Silvestri, 1927, a large Afrotropical genus with 18 species, which are mostly western African (Silvestri 1927; Golovatch et al. 2015).

The genus *Globanus* had been considered endemic to São Tomé and Príncipe until Krabbe (1982), in her global revision of Spirostreptidae, synonymized *Globanus* with *Lobogonus* Demange, 1971, and thus extended the distribution of *Globanus* to mainland western Africa. She only recognized two valid species, *G.integer* and *G.trilobatus* (Demange, 1971). The latter species is from Sierra Leone, even though Brolemann (1935), albeit cryptically inside an introductory part to his *Faune de France* monograph, had beautifully depicted the gonopod of *Aulonopygemarginescaber* (= *Globanusmarginescaber*).

In 2010, R.C. Drewes (California Academy of Sciences) collected millipedes in addition to his herpetology speciality for six weeks on São Tomé and Príncipe, but the samples consist of only one order, family, and genus: Spirostreptida, Spirostreptidae, *Globanus* Attems, 1914. *Globanusinteger* (Karsch, 1884) and *G.marginescaber* (Karsch, 1884) were both recovered on each island, along with one new species. This suggests that a *Globanus* "species swarm" exists on both São Tomé and Príncipe islands.

The present paper is a review of *Globanus*, with the description of a new species endemic to São Tomé Island. The Sierra Leone genus *Lobogonus* is revalidated and removed from synonymy under *Globanus*.

## Material and methods

This study is based on material collected in 2010 by R.C. Drewes. Some additional samples were obtained from the Muséum national d’Histoire naturelle (MNHN), Paris, France and the Royal Museum for Central Africa (MRAC), Tervuren, Belgium.

All samples are stored in 70% ethanol. Photographs were made with a Leica DFC 500 digital camera mounted on a Leica MZ16A stereo microscope. Images were processed with a Leica Application Suite program. Specimens for scanning electron microscopy (SEM) were air-dried, mounted on aluminium stubs, coated with gold and studied using a JEOL JSM-6480LV scanning electron microscope.

The terminology used to describe the gonopod conformations follows that of Hoffman (2008).

### Museum acronyms

**CAS** California Academy of Sciences, San Francisco, U.S.A.

**MNHN** Muséum national d’Histoire naturelle, Paris, France

**RMCA** Royal Museum for Central Africa, Tervuren, Belgium

**ZMB** Zoological Museum, Humboldt University, Berlin, Germany

## Systematics

### Family SPIROSTREPTIDAE

#### Genus Lobogonus

Taxon classificationAnimaliaDiplopodaSpirostreptidae

Demange, 1971

15A2ED0B-5C91-5CCF-B83F-AF1FB94D0591

##### Type species.

*Lobogonustrilobatus* Demange, 1971, by original designation.

**Figure 1.**
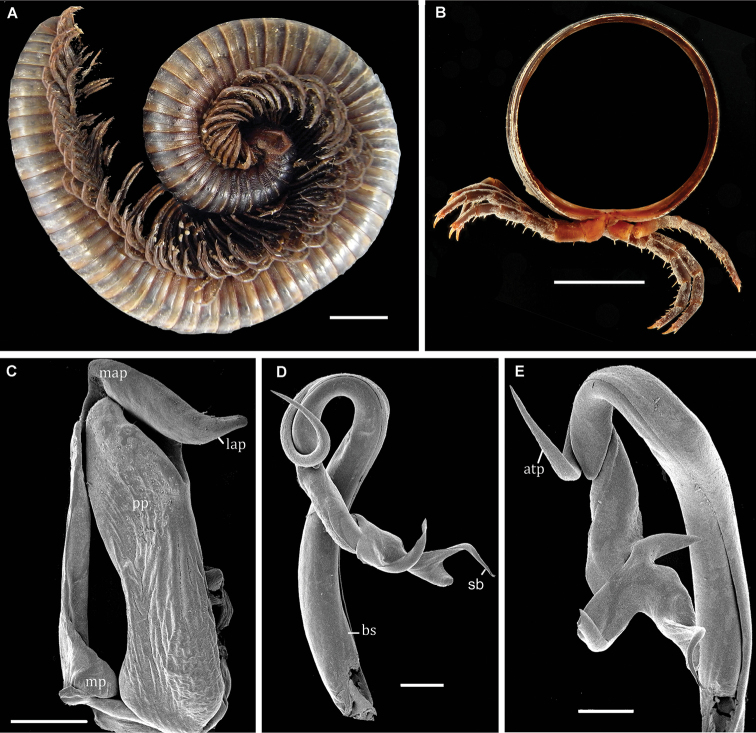
*Lobogonustrilobatus* Demange, 1971, ♂ syntype. **A** habitus, lateral view **B** transverse section of midbody segment **C** right gonopod, posterior view **D** left telopodite, anterior view **E** tip of telopodite. Abbreviations: atp = antetorsal process, bs = basomere, lap = latero-apical process, map = meso-apical metaplical process, mp = metaplica, pp = proplica, sb = seminal branch. Scale bars: 10 mm (**A**); 1 mm (**C**); 500 µm (**D**, **E**).

**Diagnosis** (after Demange 1971, with modifications). A genus of large millipedes (up to ca 200 mm long) with relatively long legs (80% of maximum body diameter). Gonocoxite stout and large, ending with a thick apicolateral projection (Fig. 1C, lap); telopodite thick and short, distally characterized by three well-differentiated lobes and a thin seminal branch (Fig. 1D, E, sb).

#### Lobogonus trilobatus

Taxon classificationAnimaliaDiplopodaSpirostreptidae

Demange, 1971

5F357C4E-DF4E-5BEA-B10F-B1491D7CB154

Figure 1

Globanus trilobatus : Krabbe 1982: 146–147.

##### Material observed.

Syntypes: 10 ♂♂, 6 ♀♀, 5 juv, Sierra Leone, Mt Loma, 02.XI.1964 (MNHM-MY-MY 6531).

##### Remarks.

**Figure 2.**
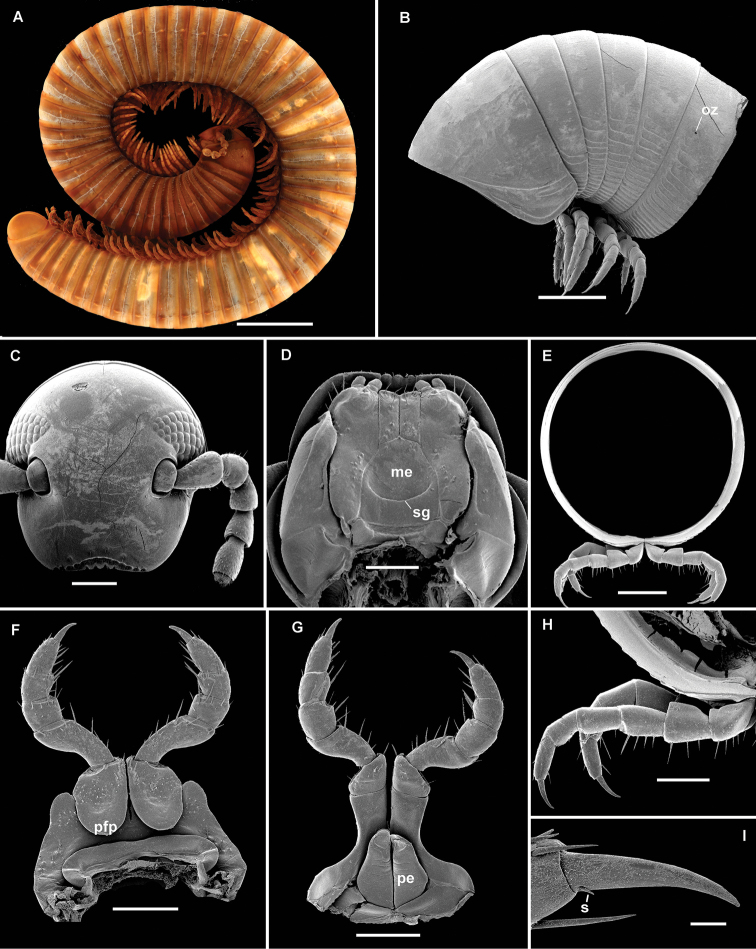
*Globanusdrewesi* sp. nov., ♂ paratype. **A** habitus, lateral view **B** collum and segments 2–6, lateral view **C** frontal view of head **D** gnathochilarium **E** midbody transversal section **F** first pair of legs, oral view **G** second pair of legs, oral view **H** midbody legs, oral view **I** midbody claw, caudal view. Abbreviations: me = mentum, pe = penes, pfp = prefemoral process, s = seta at base of claw, sg = submedian transverse groove. Scale bars: 5 mm (**A**); 1 mm (**B**) 1mm (**D, E**); 500µm (**C, D, F, G**); 200µm (**H**); 50µm (**I**).

Having studied the original description, closely examined the type species of the genus *Lobogonus* Demange, 1971 (Mt Loma region, Sierra Leone, MNHN, types), and compared its gonopods to those of *Globanus* spp., we disagree with the decision of Krabbe (1982) to merge *Lobogonus* with *Globanus*. Krabbe synonymized these two genera on account of similarities in gonopod structure, but the similarities appear to be rather superficial. In addition, both of the genera show different, totally disjunct distributions; *Globanus* is confined to São Tomé and Príncipe, whereas *Lobogonus* occurs in the Mont Loma region, Sierra Leone. *Lobogonustrilobatus* is a large millipede (ca 200 mm long) with long legs (80% of maximum body diameter; Fig. 1A, B), *vs. Globanus* species, which are considerably smaller and show relatively short legs (ca 60% of maximum body diameter; Fig. 2A, E). *Lobogonustrilobatus* has a pilose gnathochilarium, in contrast to the poorly setose one observed in *Globanus* species (Fig. 2D). Similarities of the gonopods are also superficial and only concern the post-torsal process of the gonotelopodites, with 2 or 3 lobes or processes observed in both genera. In *Globanus* these lobes are subapical lobes, whereas in *Lobogonus* they are apical. The gonocoxite of *L.trilobatus* is rather stout and large (Fig. 1C–E) compared to the slender gonocoxite observed in *Globanus* species.

#### Genus Globanus

Taxon classificationAnimaliaDiplopodaSpirostreptidae

Attems, 1914

25D35F61-48F4-5BA5-BB96-70BCC2DE2F67

##### Type species.

*Spirostreptusinteger* Karsch, 1884, by original designation.

##### Diagnosis

(after Krabbe 1982, with modifications). A genus of moderate-sized millipedes (up to 65 mm long with relatively short legs (ca 60% of maximum body diameter)). Gnathochilarium notable in that the surface sculpture of the mentum shows a submedian transverse groove (Fig. 2D, sg). Gonopod metaplica extended into an apicolateral projection, proplica slender, with or without a distolateral spine. Telopodite with a long, slender, antetorsal process, with a post-torsal process situated more distally, with 2 or 3 lobes proximal to an attenuating and slender tip.

##### Distribution.

São Tomé and Príncipe islands.

##### Species included.

*Globanusinteger* (Karsch, 1884), *G.marginescaber* (Karsch, 1884), *G.drewesi* sp. nov.

### Key to *Globanus* species

**Table:** 

1	Median lamella of telocoxite without distal proplica spine (Fig. 5B, C)	***G.integer* (Karsch, 1884)**
–	Median lamella of telocoxite with a distal proplica spine (ps)	**2**
2	Apical part of telopodite without subtriangular projection (Fig. 4C)	***G.marginescaber* (Karsch, 1884)**
–	Apical part of telopodite with a subtriangular projection (sp) (Fig. 3D, F)	***G.drewesi* sp. nov.**

#### Globanus drewesi sp. nov.

Taxon classificationAnimaliaDiplopodaSpirostreptidae

5CE51F30-9FB7-5A26-B8F6-FE4B6104871E

http://zoobank.org/6A51E9E2-E585-4003-B4AA-90CFDF04F05C

Figures 2 , 3

##### Type material.

Holotype ♂, Republic of São Tomé and Príncipe, São Tomé Island, Morro Provaz Ridge, road to Lagoa Amelia, alt. 1475 m, 3.III.2010, 0°16’58”N, 6°35’12.5”E, Bob Drewes leg. (CASENT9032626).

Paratypes: 1 ♂, 6 ♀♀, Republic of São Tomé and Principe, São Tomé Island, Morro Provaz Ridge, Headwaters of Rio do Oro, alt. 1240 m, 3.III.2010, 0°17’3.8”N, 6°35’57.5”E, Bob Drewes leg. (CASENT9032626).

##### Etymology.

Honours Bob Drewes, the collector.

##### Diagnosis.

Distinguished from other species of the genus by the acuminate distal prolongation of the gonopod proplica. Post-torsal process of telopodite with two attached lamellae; apex rotated 360°ending in a tongue-shaped process.

##### Description.

Holotype, adult male with 52 body rings (including preanal ring), length ca 65 mm (curved and broken), maximum body diameter 4.23 mm.

Colour (in alcohol) predominantly brownish; prozonae yellowish brown; metazonae, legs, and antennae dark brown.

Head without modifications, smooth and polished, interantennal isthmus 1.11 mm, interocellarial isthmus 1.15 mm; antennae short (55% of maximum body diameter), extending up to posterior edge of collum; sensory pits on antennomeres 5 and 6 present, on 5^th^ antennomere smaller. Eyes reniform, ommatidia arranged in five series: 9-8-7-6-4 = 34, counted from behind (Fig. 2C). Gnathochilarium with a transverse row of about 10 setae along distal margin and 6 large setae on each lingual lamella; mentum elongate-triangular with a few setae and a well-marked submedian transverse groove (Fig. 2D, sg).

Collum subcylindrical, smooth, lateral ends with three submarginal striae (Fig. 2B).

Body rings circular (height/width ratio of midbody rings 0.98–1.0), no legless body rings in front of telson. Prozonae smooth, suture between pro- and metazonae straight. Metazonae equal in diameter to prozonae; metazonital striae present below ozopore level; ozopores starting with segment 6, rather vague, located just behind suture on midbody segments (Fig. 2B, oz). Paraprocts convex, distal margins set off by a submarginal groove. Hypoproct not fused to preceding segment.

First pair of male Iegs as shown in Fig. 2F, with 5 or 6 setose tubercles laterally; each prefemur with a prominent basal projection on anterior side and with short setae medially. Second pair of legs and penes as in Figure 2E. Walking legs rather short (length 61% of midbody diameter, only tarsi visible from above when stretched; Fig. 2E, H), distal third with ventral tibial pads extending to proximal third of tarsi. Tarsal claws long and curved, each with a small basal seta (Fig. 2I, s).

**Figure 3.**
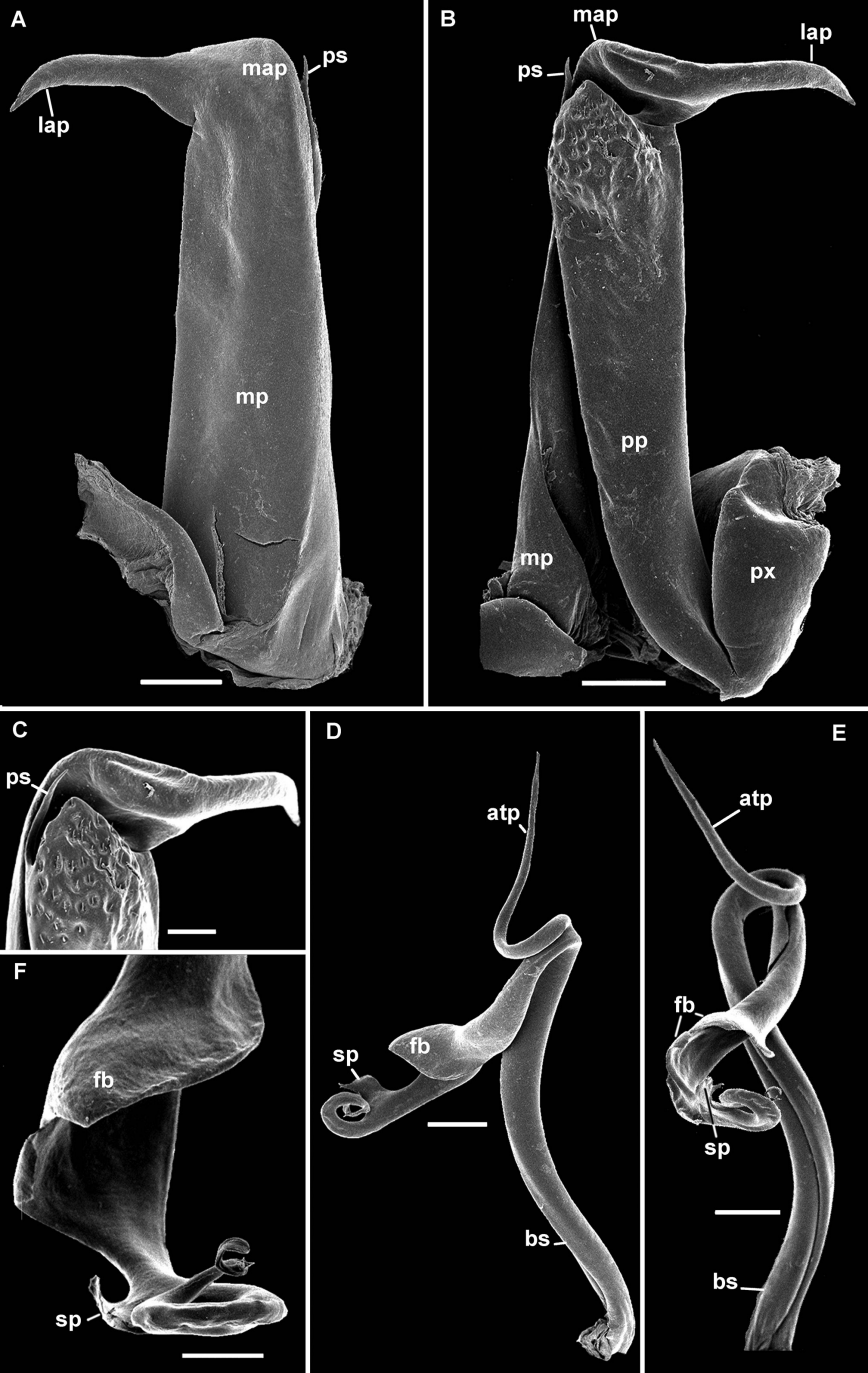
*Globanusdrewesi* sp. nov. ♂ paratype. **A, B** left coxite, oral and caudal views, respectively **C** tip of left coxite, caudal view **D, E** left telopodite detached, anterior and posterior views, respectively **F** tip of left telopodite, posterior view. Abbreviations: atp = antetorsal process, bs = basomere, fb = flattened blade, lap = latero-apical process, map = meso-apical metaplical process, mp = metaplica, pp = proplica, ps = proplica spine, px = paracoxite, sp = subtriangular projection. Scale bars: 200 µm (**A**, **B**, **D**, **E**); 100 µm (**C**, **F**).

Gonopods with a small sternum; proplica (pp) slender, with an acuminate distolateral spine and a field of short setae proximal to it. Metaplica (mp) slender proximally, expanded distally to form a latero-apical metaplical process (lap), slightly projecting outside the body when at rest (Fig. 3A–C). Telopodite as shown in Figure 3D, E, placed on anterior side of gonopod, antetorsal process (atp) long and slender, originating near arculus. Torsate region comprising less than half of telopodite length, distal third giving rise to a flattened blade (fb) and, beyond this, to a subtriangular projection (sp) (Fig. 3D). Apex rotated 360° ending in a tongue-shaped process. Prostatic groove running straight to tip of solenomere, ending just before the tongue-shape process level to a small digit (Fig. 3F).

Paratype male agrees with holotype in all structural details, but females larger (up to 80 mm in length and ca 6 mm in diameter), with short legs (ca 60% of midbody diameter) devoid of tibial pads; the number of body rings also varies between specimens (up to 56 body rings including preanal ring). Vulvae placed vertically inside segment 3; no setae on vulvae.

The other characters agree with those of the holotype.

##### Distribution.

The species seems to be endemic to São Tomé Island.

##### Relationships.

Although the three species of *Globanus* are externaly very similar, the gonopod structure suggests that the new species is closer to *G.marginescaber*. In both species, the median lamella of the telocoxite shows a well-marked distal proplica spine, which is absent in *G.integer*.

#### Globanus marginescaber

Taxon classificationAnimaliaDiplopodaSpirostreptidae

(Karsch, 1884)

66F0AC80-9257-565C-B411-8B11FD9AD92D

Figure 4

Spirostreptus (Nodopyge) marginescaber Karsch 1884: 58–59
Aulonopyge marginescaber Brolemann 1935: 63.
Globanus marginescaber : synonymy after Spelda (1993).

##### Material examined.

Type material: not *Globanusmarginescaber*, revised.

Other material: 13 ♂♂, 15 ♀♀, 1 juv., Príncipe Island, Bela vista, alt. 40 m, 1°37’10.8”N, 7°24’49.7”E, 9.II.2010, B. Drewes leg. (CAS 9032625); 6 ♂♂, 14 ♀♀, Principe, E side, road to Infante Henrique, alt. 115–150 m, 1°36’2.4”N, 7°24’56”E, 9.II.2010, B. Drewes leg. (CAS 9032624); 1 ♂, Príncipe, E side, road to Infante Henrique, alt. 115–150 m, 1°36’2.4”N, 7°24’56”E, 9.II.2010, B. Drewes leg. (CAS 9032437); 2 ♂♂, 7 ♀♀, Príncipe, slope of Pico Papagaio, alt. 315–550 m, 1°37’10”N, 7°23’28”E, 8.III.2010, B. Drewes leg. (CAS 9032621); 1 ♂, São Thomé, Morro Provaz Ridge, headwaters of Rio do Oro, alt. 1240 m, 0°17’38.8”N, 6°35’57.5”E, 3.III.2010, B. Drewes leg. (CAS 9032483); 1 ♂, Principe, Bela vista, alt. 40 m, 1°37’10.8”N, 7°24’49.7”E, 11.III.2010, B. Drewes leg. (CAS 9032482); 1 ♂, Príncipe, Morro Provaz ridge, alt. 1275 m, 0°7’20.9”N, 6°35’50.9”E; 5.III.2010, B. Drewes leg. (CAS 9032432); 1 ♂, Príncipe, slope of Pico Papagaio, alt. 315–550 m, 1°37’10”N, 7°23’28”E, 8.III.2010, B. Drewes leg. (CAS 9032488).

##### Diagnosis.

Differs from *G.integer* by the presence of a distal proplica spine and from *G.drewesi* by the absence of a subtriangular projection in the distal third of the telopodite.

##### Description

(based on specimens CAS 9032625). Length of males ca 55 mm, width of midbody metazonae ca 0.5 mm, length of females ca 55 mm, width of midbody ca 5 mm. Colour in alcohol brown, prozonae usually light brown; antennae and clypeolabral region light yellow-brown; venter and legs yellowish.

**Figure 4.**
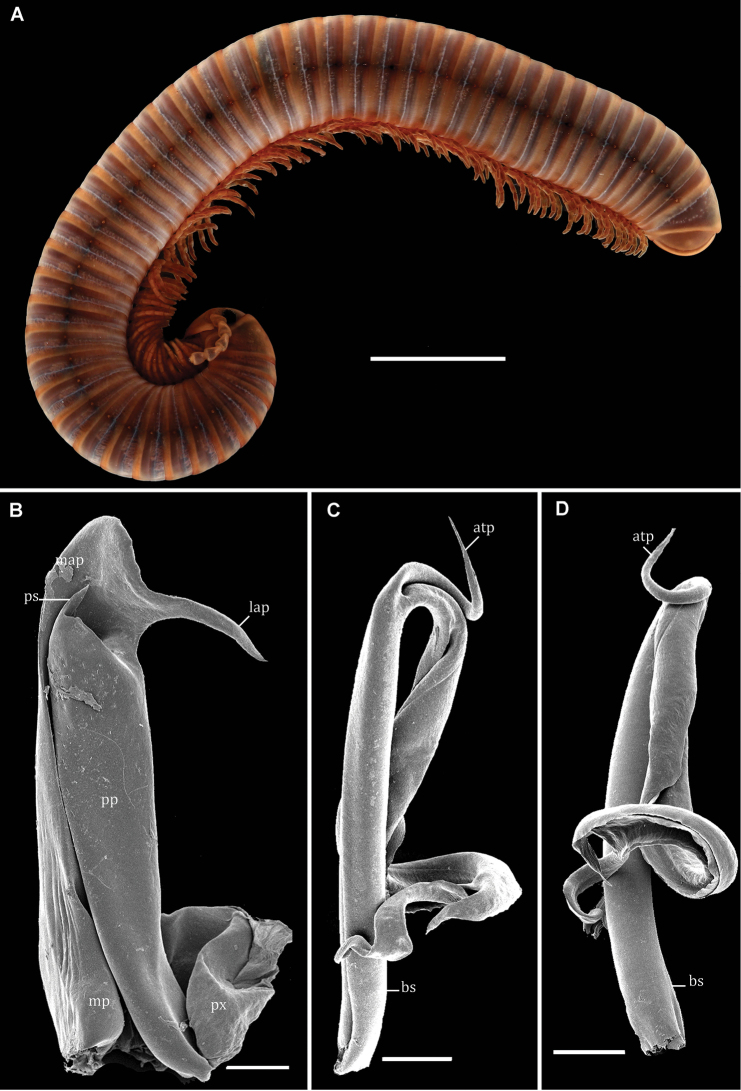
*Globanusmarginescaber* (Karsch, 1884), ♂ topotype from Príncipe Island. **A** habitus, lateral view **B** left coxite, posterior view **C, D** right telopodite, posterior and mesal views, respectively. Abbreviations: atp = antetorsal process, lap = latero-apical process, , map = mesapical metaplical process, mp = metaplica, pp = proplica, ps = proplica spine, px = paracoxite. Scale bars: 5 mm (**A**); 200 µm (**B**, **C**, **D**).

Somatic characters as in previous species (Fig. 4A).

Gonopods with a small sternum; proplica slender, with an acuminate distolateral spine and a field of short setae proximal to it. Metaplica slender proximally, expanded distally to form a lateral cone, slightly projecting outside body when at rest (Fig. 4B). Telopodite as shown in Figure 4D, placed on anterior side of gonopod; antetorsal process long and slender, originating near arculus. Torsate region comprising approximately half of telopodite length, proximal third giving rise to an acute lateral process; beyond this, telopodite slightly flattened and attenuating regularly towards tip (Fig. 4C). Prostatic groove running straight to tip of solenomere, ending just before telopodite apex.

##### Remarks.

This species was originally described so poorly by Karsh (1884) from the female holotype that Attems (1914) considered it as a species *incertae sedis*. The holotype is still in the Berlin Museum (ZMB), but its revision reveals that actually it belongs to the family Harpagophoridae and is definitely not the true type of *marginescaber*. This was already observed by Richard Hoffman (pers. comm.), who suggested some mixing of labels, which must have occurred when the collection was re-organized. The first illustration of the gonopods was published in a text book by Brolemann (1935: 63), simply to show the complex conformation of a spirostreptid gonopod. Krabbe (1982), in her revision of the family Spirostreptidae, did not mention the species, but Spelda (1993) did and provided new drawings of the gonopods of both *Globanus* species then known from São Tomé and Príncipe. The gonopod structure of the specimen observed here agrees with the drawing presented by Spelda (1993) for *G.marginescaber*, except that our male shows no small spine beyond the lateral process. Another difference is the body size; the samples from the MNHN are much larger compared to the specimens collected by R.C. Drewes: males to only 11 cm long (maximum body diameter 0.9 cm) and females to 13.5 cm long (maximum body diameter 1 cm).

This species occurs both on São Tomé and Príncipe.

#### Globanus integer

Taxon classificationAnimaliaDiplopodaSpirostreptidae

(Karsch, 1884)

73EF669F-6004-5AAA-9517-DF5C5D72FCD3

Figure 5

Spirostreptus (Nodopyge) integer Karsch 1884: 57–58
Spirostreptus (Nodopyge) molleri Verhoeff 1892: 193–199
Scaphiostreptus molleri Attems 1914: 39
Rhopaloditius molleri Verhoeff 1938: 20
Globanus molleri Attems 1950: 188
Globanus integer : synonymies after Demange (1970).

##### Material examined.

Type material: 1 ♂ syntype, São Thomé, Greeff leg. (ZMB 933).

Other material: 1 ♂, 1 ♀, São Thomé, Neves, 0°22’N, 06°34’E, in litter, cocoa plantation, 27.X.1999, W. Tavernier leg. (MRAC 18.531); 2 ♂♂, 8 ♀♀, 2 juv., São Thomé, Morro Provaz Ridge, alt. 1275 m, 0°17’20.9”N, 6°35’50.9”E, III.2010, B. Drewes leg. (CAS 9032433); 1 ♂, São Thomé, Nova Moca, alt. 920 m, 0°17’25.8”N, 6°37’58.1”E, 27.II.2010, B. Drewes leg. (CAS 9032435); 1 ♂, 1 ♀, São Thomé, Nova Moca, alt. 920 m, 0°17’25.8”N, 6°37’58.1”E, 27.II.2010, B. Drewes leg. (CAS 9032485); 8 ♀♀, São Thomé, Nova Moca, alt. 900 m, 0°17’25.8”N, 6°37’58.1”E; 27.II.2010, B. Drewes leg. (CAS 9032434); 2 ♂♂, 1 ♀, São Thomé, Nova Moca, alt. 900 m, 0°17’25.8”N, 6°37’58.1”E, 27.II.2010, B. Drewes leg. (CAS 9032436); 4 ♀♀, São Thomé, Nova Moca, alt. 920 m, 0°17’25.8”N, 6°37’58.1”E, 27.II.2010, B. Drewes leg. (CAS 9032484); 2 ♂♂, 8 ♀♀, 1 juv., São Thomé, Nova Moca, alt. 920 m, 0°17’25.8”N, 6°37’58.1”E, 27.II.2010, B. Drewes leg. (CAS 9036824).

##### Diagnosis.

Differs from congeners by the absence of a distal proplica spine, telopodite distally stout and with attached, large, plate-like lamellae.

##### Remark.

**Figure 5.**
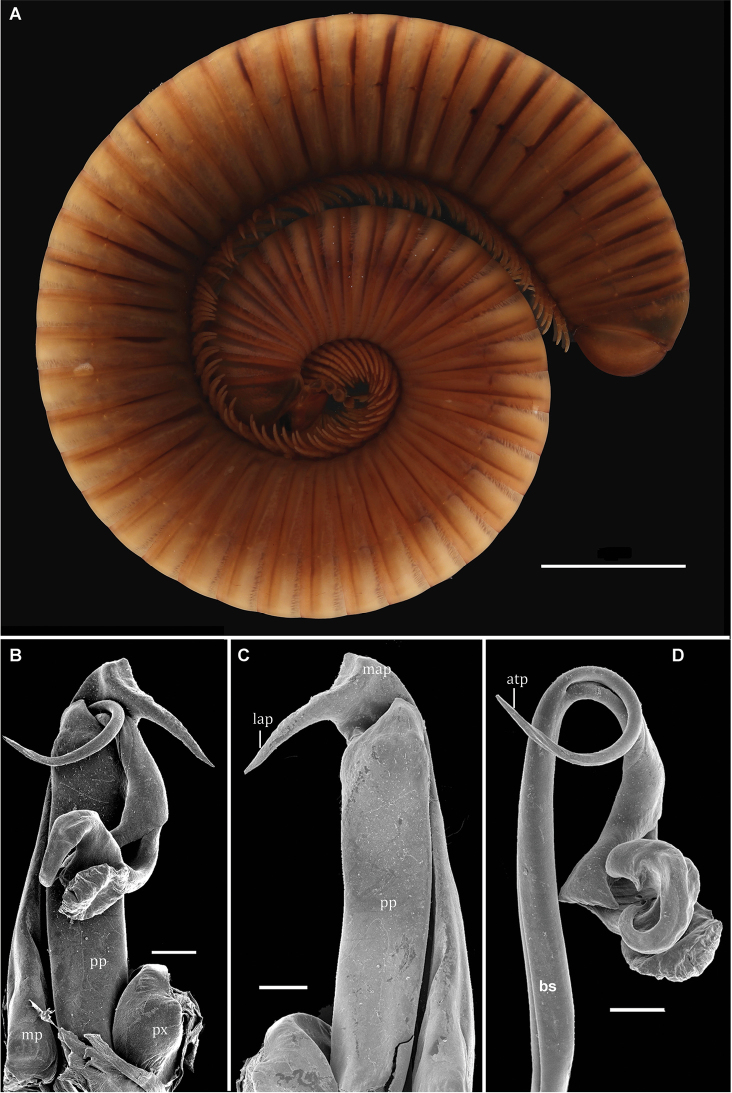
*Globanusinteger* (Karsch, 1884), ♂ topotype. **A** habitus, lateral view **B** right gonopod, posterior view **C** left coxite, posterior view **D** right telopodite, posterior view. Abbreviations: atp = antetorsal process, bs = basomere, lap = latero-apical process, map = meso-apical metaplical process, mp = metaplica, pp = proplica, ps = proplica spine, px = paracoxite. Scale bars: 5 mm (**A**); 200 µm (**B**–**D**).

The gonopod structure of a topotype (Fig. 5) agrees closely with the drawings presented by Spelda (1993).

This species seems to be restricted to São Tomé Island.

## Conclusion

*Globanustrilobatus*, originally described in the monotypic *Lobogonus* (Demange 1971) and then relegated to *Globanus* by Krabbe (1982), is returned to *Lobogonus* with that genus revalidated. A close examination of type specimens of *L.trilobatus* (in the MNHN) shows that species of these two genera are strikingly different both in habitus and gonopod structure and also have completely disjunct distributions. Thus, there is clear support for the separation of *Globanus* and *Lobogonus*.

**Figure 6.**
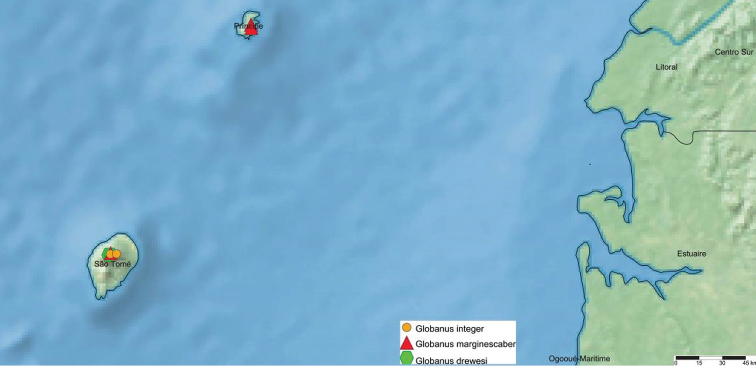
Distribution of *Globanus* species in São Tomé and Príncipe.

The oligotypic genus *Globanus* currently encompasses three species and seems to be endemic to São Tomé and Príncipe islands. Among the species, only *G.marginescaber* occurs on both islands, while *G.integer* and *G.drewesi* sp. nov. apparenty are restricted to the larger São Tomé Island (Fig. 6). The trio may well be regarded as another example of “insular species swarms” among Diplopoda, however small, much like several others long reported from all main archipelagos of Macaronesia, as well as the Hawaiian Islands and New Caledonia (e.g. Enghoff 1992, 1993).

## Supplementary Material

XML Treatment for Lobogonus

XML Treatment for Lobogonus trilobatus

XML Treatment for Globanus

XML Treatment for Globanus drewesi

XML Treatment for Globanus marginescaber

XML Treatment for Globanus integer
